# Survival and Predictors of Mortality of Congenital Diaphragmatic Hernia in Newborns at a Tertiary Care Hospital in Saudi Arabia

**DOI:** 10.7759/cureus.54364

**Published:** 2024-02-17

**Authors:** Khalid Al-Shareef, Mohammed Bhader, Mohammed Alhindi, Khalid Helmi, Salman Ashour, Ahmed Moustafa, Abdullah Al-Harbi, Amir Abushouk, Mansour A AlQurashi

**Affiliations:** 1 College of Medicine, King Saud Bin Abdulaziz University for Health Sciences, Jeddah, SAU; 2 Pediatrics, Ministry of National Guard Health Affairs, Jeddah, SAU; 3 Research and Development, King Abdullah International Medical Research Center, Jeddah, SAU; 4 College of Medicine, King Saud bin Abdulaziz University for Health Sciences, Jeddah, SAU; 5 Neonatology, Ministry of National Guard Health Affairs, Western Region, Jeddah, SAU; 6 Pediatric, Neonatology Division, King Abdulaziz Medical City, Ministry of National Guard Health Affairs, Jeddah, SAU

**Keywords:** prevalence, mortality rate, predictor tools, cdh, congenital diaphragmatic hernia

## Abstract

Background

Congenital diaphragmatic hernia (CDH) is a condition where abdominal contents protrude into the chest due to defects in the diaphragm muscle. It is considered an emergency that needs urgent intervention to prevent further complications or death. Our study aimed to estimate survival and evaluate predictors of mortality in newborns with CDH using available prediction tools in the literature.

Methods

This retrospective cohort study included neonates with CDH in King Abdulaziz Medical City (KAMC), Jeddah, from 2000 to 2021. Prevalence, demographics, and clinical characteristics were compared between surviving and deceased infants. C-statistics were used to measure the area under the curve for the prenatal and postnatal predictor tools, and a p-value of <0.05 was considered significant.

Results

Between 2000 and 2021, 45 neonates with CDH were included (six per 10,000 inborn live births). The mortality rate was 51.1%. The differences in demographics were not significant among surviving and deceased patients. One prenatal predictor tool, the lung-to-head ratio, was found to be significant; in addition, three postnatal predictor tools of mortality, SNAP-II, CDHSG-probability survival, and Brindle Score, had the highest concordance (C) statistics of 0.8, 0.79, and 0.8, respectively.

Conclusion

Although the incidence of CDH was found to be higher in our study compared to global statistics, our mortality rates correspond with international figures. The most significant differences between predictors and prediction models of mortality were lung-to-head ratio prenatally, SNAP-II, CDHSG-probability survival, and Brindle Score postnatally. Further multicentered studies are recommended with a larger sample size.

## Introduction

Congenital diaphragmatic hernia (CDH) is a condition in which the contents of the abdomen protrude into the thoracic cavity due to a defect that occurs during the critical phase of the development of the bronchial branching and pulmonary artery in utero, which also affects the proper development of the diaphragm, affecting lung development. The defect can vary in presentation, ranging from a minor narrowing of the rims of the posterior diaphragmatic muscles to complete agenesis of the diaphragm [1-3]. CDH, which is considered a physiological emergency, is a rare condition that occurs in approximately 0.031% of all live births [4]. A recent systematic review was conducted to estimate the prevalence of CDH worldwide, and it estimated an incidence of 2.3 in 10,000 [5]. Some genes and genetic disorders have been linked with this disorder despite the fact that the cause of most cases of CDH is unknown. Furthermore, it is believed that the cause might be multifactorial, involving genetic factors, environmental factors, and nutritional deficiency [6-9]. Primary pulmonary hypertension and pulmonary hypoplasia are two factors that increase the risk of mortality and morbidity of CDH; moreover, CDH is associated with anomalies that are related to the cardiovascular, gastrointestinal, genitourinary systems, and trisomy. Therefore, CDH is considered to be one of the most difficult congenital diseases to manage [7-10]. Various risk predictor tools for mortality used by clinicians are well set to produce or affirm an individualized risk profile for CDH patients, evaluating them from gestation until the early beginning of the postnatal period. These predictor tools are very important for the thorough and efficient management of CDH [11]. To our knowledge, valid risk predictor tools for CDH have not been used in any of the health centers in Saudi Arabia. The rationale for this study is to establish benchmark scores that provide clinicians and parents with a general estimate of newborns who are presented with CDH, to allocate resources based on patient condition, to establish a medical conditional role based on scores that determine mortality, and to address the lack of medical resources that can facilitate the management of CDH. This study aimed to estimate the incidence and mortality rate and use predictors of mortality and prediction models in newborns with CDH using available prediction tools in the literature.

## Materials and methods

This research was conducted at the Department of Pediatrics, division of neonatology, King Abdelaziz Medical City, Jeddah, Western Region, Saudi Arabia. The inclusion criteria are: All life births from 1/1/2000 to 31/12/2021 who were diagnosed with CDH regardless of the time of diagnosis (antenatal or postnatal), type, size, or site of the diaphragmatic defect were included in the study. There were no exclusion criteria. The study design was a retrospective cohort study. Furthermore, the sampling was a non-probability consecutive sampling technique.

Data were extracted by authors from Bestcare® software (Electronic Health Records) along with physical records of hospital admissions using a pre-specified data collection Excel sheet. Data targeted were as follows: patients' demographics, co-morbidities, maternal data, defect size, head ratio (%LH) data used for the snap-II score, brindle score, and congenital diaphragmatic hernia survival group (probability of survival) (CDHSG-PS). Later, data was entered into statistical IBM Corp. Released 2019. IBM SPSS Statistics for Windows, Version 26.0. Armonk, NY: IBM Corp. for analysis.

For the definition of lung to head ratio (%LH), CDHSG-PS, Brindle Score, SNAP-II, and congenital diaphragmatic hernia defect size (CDHSG Defect Size) (Appendix 1).

Data collection and analysis

Data was collected into the data collection sheet and then extracted into IBM Corp. Released 2019. IBM SPSS Statistics for Windows, Version 26.0. Armonk, NY: IBM Corp. for data mining, coding, and management, followed by statistical analysis. Patients’ demographics and characteristics were analyzed as follows: Categorical data was described using frequencies, while measured data were analyzed utilizing means and standard deviations or median and interquartile range based on normality. Predictors of mortality were calculated as per Tim Jancelewics and Mary E. Brindle (Appendix 1). Measures of accuracy were calculated by the standard methods of 2x2 tables as well as the 95% CI. Concordance statistics (C-statistics), which is a measure of the quality of fit for binary outcomes in a logistic regression model, was used to calculate the predicted mortality. Furthermore, C-statistics was adjusted for unavailable data.

## Results

Maternal and newborn background characteristics 

Table 1 summarizes maternal and infant characteristics. This study estimated the prevalence of CDH between 2000 and 2021. During this period, the total number of births in King Abdulaziz Medical City in Jeddah was estimated to be 66,844. Out of the total number of births, 36 inborns were diagnosed with CDH. Therefore, the incidence of CDH in King Abdulaziz Medical City was 1 per 1856 (6 per 10,000) live inborns. In addition to the 36 inborn, this study included nine infants who were born outside of the center and admitted as cases of CDH. In total, 45 (36 born in house and another nine referred) neonates with CDH were included in this study, 34 (75.6%) males and 11 (24.4%) females. The mortality rate was 23 (51.1%), where 19 infants died preoperatively (42.2%) (15 males, four females), and four died postoperatively (8.8%) (four males). In addition, 20 infants were stable enough to get surgery, and four of them died (20%). The median number of days till death for neonates who died was two days (1-9). The remaining 22 infants (48.8%) (19 males, three females) survived. The median length of stay for infants who survived was 18 (10-25) days. The mean age of mothers whose infants died was 30.23 (5.88±), and a mean of 28.18 (6.12±) for those whose infants survived. The BMI of mothers whose infants died was 28.75 (6.78±), and for those whose infants survived, it was 28.58 (6.24±). The modes of delivery among those who died were 12 vaginal deliveries (SVD) and 11 C/S. The modes of delivery among those who survived were 12 SVD and 9 C/S. Mothers’ gravidity of deceased infants was three (2-6) and two (1-3) for those who were alive. The mothers’ parity of dead infants was two (1-3) and was one (1-4) for those who were alive. 

Consanguinity among parents of dead patients was 11 (68.7%) and five (31.3%) for those who survived. Nineteen (82.6%) were booked among infants who died, and 13 (81.0%) were booked among infants who lived. Twenty-two (95.65%) were inborn among those who died, and 14 (63.6%) among those who survived. Being antenatally diagnosed, 17 (77.3%) of the infants died and nine (60%) lived. The mean GA at birth among patients who died was 37.33 (1.82±), and a GA of 38.21 (2.32±) among patients who lived. The GA at diagnosis for dead infants was 30.59 (7.51±) and 32.79 (5.89±) for surviving infants. Four (23.5%) of the patients who died had associated anomalies, while one (11.1%) of those who lived had an associated anomaly. Among those who died, four (25%) had ECHO prenatally and 15 (65.2%) postnatally; furthermore, one (9.1%) who survived had ECHO prenatally and 17 (85%) had it done postnatally. One patient underwent ECMO. Among those who died, four (25%) performed an MRI, and two (20%) among infants who lived did so. Apgar score (5 min) among patients who died was seven (6.5-9) and nine (5-8) among those who lived. The mean BW among deceased patients was 274 (0.66±) and 2.89 (0.63±) among surviving patients. 

Descriptive analysis 

Fisher Exact Test n (%), T independent test and mean (SD), and Mann-Whitney & median (IQR) were utilized to analyze the p-value. [MSAB3] Maternal age showed statistical insignificance (p = 0.29) in the survival rate. Survival between males and females was not significant (p = 0.17), with an odds ratio of 0.29. BMI showed statistical insignificance (p = 0.93) in mortality. Maternal comorbidity among survivors and non-survivors was insignificant (p = 0.89); among non-survivors, 81% did not have maternal comorbidities, 9.5% had gestationally related comorbidities, and 9.5% had chronic comorbidities. The mode of delivery did not show significance (p = 0.74). Delivery complications were insignificant (p = 0.49), with an odds ratio of 1.22. Gravidity and parity among survivors and non-survivors demonstrated statistical insignificance (p < 0.15) and parity (p = 0.09), respectively. Consanguinity did not show any statistical significance (p = 0.23), with an odds ratio of 2.75. Booking status was not significant (p = 0.88) on survival, with an odds ratio of 1.1. In/outborn demonstrated significance in predicting the mortality of CDH patients (p < 0.01), with an odds ratio of 0.08. The time of diagnosis antenatally and postnatally was insignificant (p = 0.29), with an odds ratio of 2.2. Gestational age at birth and at diagnosis did not demonstrate significance (p = 0.32/0.36) for survival. The presence of an associated anomaly was not significant (p = 0.62), with an odds ratio of 2.46. ECHO performed prenatally and postnatally, both of which did not show significant (p = 0.61) or (p = 0.17). Performing the ultrasound demonstrated insignificance (p = 0.12). MRI showed insignificance (p = 0.52), with an odds ratio of 1.3. The lowest temperature did not show significance (p = 0.71) in predicting mortality. The P/F ratio demonstrated large significance (p < 0.001) in predicting survival. The APGAR score (5 min) showed great statistical significance (p < 0.001). Birth weight was statistically not significant (p = 0.47). The mean blood pressure was statistically insignificant (p = 0.52). The lowest serum pH demonstrated high significance in predicting mortality (p <0.001). Urine output was statistically significant (p = 0.003). Both ABG (PCO2) during the first hour and the first 24 hours demonstrated excellent significance statistically (p < 0.011) and (p <0.006), respectively (Table 1).

**Table 1:** Maternal and neonates background characteristics *n (%); ** Mean (SD); *** Median (IQR). N: number of patients, ECHO: Echocardiogram, ECMO: Extracorporeal membrane oxygenation, MRI: Magnetic resonance imaging, PaO2: Arterial oxygen tension, FiO2: Fractional inspired oxygen, ABG: Arterial blood gas.

	Total (n=45), n (%)	Death (n=23(51.1)), n (%)	Alive (n=22(48.9)), n (%)	P-value
Maternal characteristics				
** Age	29.33 (6±)	30.23 (5.88±)	28.18 (6.12±)	0.29
** Body Mass Index	28.7 (6.43±)	28.75 (6.78±)	28.58 (6.24±)	0.93
* Comorbidities	Missing 6 (13.3%)			0.89
Not present	32 (71.1%)	17 (81%)	15 (83.3%)	
Gestational Related	3 (6.7%)	2 (9.5%)	2 (11.1%)	
Chronic	4 (8.9%)	2 (9.5%)	1 (5.6%)	
*Delivery Mode	Missing 1 (2.2%)			0.74
Vaginal Delivery	24 (53.4%)	12 (52.2%)	12 (57.1%)	
Cesarean section	20 (44.4%)	11 (47.8%)	9 (42.9%)	
*Delivery complication	Missing 8 (17.8%)			0.491
Yes	2 (4.4%)	2 (10%)	0	
No	35 (77.8%)	18 (90%)	15 (100%)	
*** Gravidity	3 (2-5.5)	3 (2-6)	2 (1-3)	0.15
*** Parity	1 (1-3)	2 (1-4)	1 (0-2)	0.094
*** Abortion	0 (0-1)	0 (0-1)	0 (0-1)	0.49
*Consanguinity	Missing 20 (44.4%)			0.23
Yes	16 (35.6%)	11 (73.3%)	5 (50%)	
No	9 (20%)	4 (26.7%)	5 (50%)	
*Antenatal Care	Missing 1 (2.2%)			0.88
Followed up	36 (80%)	19 (82.6%)	17 (81.0%)	
No records	8 (17.8%)	4 (17.4%)	4 (9%)	
*In/Outborn status				0.007
Inborn	36 (80%)	22 (95.65%)	14 (63.6%)	
Outborn	9 (20%)	1 (4.35%)	8 (36.4%)	
*Time at Diagnosis	Missing (8 (17.8%)			0.29
Postnatal	11 (24.4%)	5 (22.7%)	6 (40%)	
Antenatal	26 (57.8%)	17 (77.3%)	9 (60%)	
Neonatal characteristics				
*Gender				0.17
Male	34 (75.6%)	15 (65.2%)	19 (86.4%)	
Female	11 (24.4%)	8 (34.8%)	3 (13.6%)	
**Gestational Age at Diagnosis	31.4 (6.9±)	30.59 (7.51±)	32.79 (5.89±)	0.36
*Associated Anomaly	Missing 19 (42.2%)			0.62
Yes	5 (11.1%)	4 (23.5%)	1 (11.1%)	
No	21 (46.7%)	13 (76.5%)	8 (88.9%)	
*Prenatal ECHO	Missing 18 (40%)$			0.61
Yes	5 (11.1%)	4 (25%)	1 (9.1%)	
No	22 (48.9%)	12 (75%)	10 (90.9%)	
*Postnatal ECHO	Missing 2 (4.4%)			0.175
Yes	32 (71.1%)	15 (65.2%)	17 (85%)	
No	11 (24.4%)	8 (34.8%)	3 (15%)	
*Ultrasound				0.12
Yes	30 (66.7%)	18 (78.2%)	12 (54.5%)	
No	15 (33.3%)	5 (21.8%)	10 (45.5%)	
*ECMO				-
Yes	1 (100%)	1 (100%)	0	
No	0	0	0	
*MRI	Missing 19 (42.2%)			0.52
Yes	6 (13.3%)	4 (25%)	2 (20%)	
No	20 (44.4%)	12 (75%)	8 (80%)	
**Lowest Temperature	35.9 (1.7±)	35.78 (1.67±)	35.98 (1.72±)	0.71
**P/F Ratio (PaO2/FiO2)	130.9 (91.9)	76.77 (49.66±)	184.95 (93.38±)	<0.001
***Apgar Score (5 min)	8 (6.5-9)	7 (5-8)	9 (8-9)	<0.001
**Birth Weight (kg)	2.81 (0.65±)	2.74 (0.66±)	2.89 (0.63±)	0.47
**Mean Blood Pressure	42.5 (6.7±)	41.8 (8.27±)	43.16 (4.89±)	0.52
**Lowest serum pH	7.14 (0.24±)	6.99 (0.25±)	7.29 (0.08±)	<0.001
**Urine Output	2.46 (1.76±)	1.53 (1.47±)	3.21 (1.64±)	0.003
**ABG (PCO_2_ during 1^st^ hour)	56.9 (15.92±)	62.66 (16.43±)	49.19 (11.75±)	0.011
**ABG (PCO_2_ during 1^st^ 24 hours)	50.72 (24.46±)	61.22 (29±)	39.64 (11.14±)	0.006

Prenatal predictor tools of mortality in CDH are presented in Table 2, in which nine outborn and five inborn patients had no data to include in the analysis. The lung-to-head ratio had an excellent C-statistics with a value of 0.84, in which the mean ratio (SD) in dead patients was 0.89 (0.55±), while in alive patients, it was 1.5 (0.42±), and the p-value was significant (0.024). For patients who had stomach herniation, the C-statistics was found to be statistically insignificant, with a p-value of 0.9. The median (IQR) shows that seven (43.7%) of them died while seven (58.3%) survived; in comparison, for patients who did not have any stomach herniation, nine (56.3%) of them have died while five (41.7%) have survived. For infants who had liver herniation, the C-statistics of liver herniation was statistically insignificant as well, with a p-value of 0.67. The median (IQR) shows that four (23.6%) of them have died while four (36.4%) have survived; patients who, on the other hand, did not have any liver herniation, 13 (76.4%) have died, while seven (63.6%) have survived. 

**Table 2:** Prenatal predictor tools of mortality in congenital diaphragmatic hernia *Fisher’s Exact Test and n (%); ** T independent test and Mean (SD), C: C-statistics.

	Outcome	Death	Alive	Best C-statistics	P-value
** Lung-to-head ratio	Survival	0.89 (0.55±)	1.5 (0.42±)	C 0.84 (Our study 2023) C 0.73 (Keller 2003) C 0.7 (Bebbington 2014)	0.024
*Stomach Herniation	Survival			C 0.83 (Basta 2016) C 0.72 (Gentili 2015) C 0.49 (Our study 2023)	0.9
Yes		7 (43.7%)	7 (58.3%)		
No		9 (56.3%)	5 (41.7%)		
*Liver Herniation	Survival			C 0.57 (Our study 2023)	0.67
Yes		4 (23.6%)	4 (36.4%)		
No		13 (76.4%)	7 (63.6%)		

The postnatal predictor tools of mortality in CDH are presented in Table 3. SNAP-II was a significant predictor with a C-statistics of 0.8 and a p-value of 0.007, in which the mean (SD) in deaths was 18 (8.50±), while in survival, it was 7.62 (8.8±). CDHSG-probability survival was also significant with a C-statistics of 0.79; moreover, the p-value was significant (0.003) with a median (IQR) for dead patients of 65.5 (84-83), while in alive patients, it was 83.5 (76.5-88). CDHSG Defect Size also had a significant predictive value with a C-statistics of 0.69, but the p-value is insignificant (0.307) with n (%) for defect size A deaths were one (25%), and survival was seven (43.8%). For defect size B, there were no deaths, and there was a survival rate of five (31.2%). For defect size C, deaths were three (75%), and survivals were four (25%). And for defect size D, there were no cases. The Brindle Score was significant in terms of a C-statistics of 0.80 and a p-value of 0.003, with a mean (SD) of 2.36 (1.29±) in deceased patients and 0.93 (1±) for survived patients.

**Table 3:** Postnatal predictor tools for mortality in congenital diaphragmatic hernia *Fisher’s exact test and n (%); ** T independent test and mean (SD); *** Mann-Whitney Test and median (IQR). CDHSG: Congenital diaphragmatic hernia study group, SNAP: Scores for neonatal acute physiology.

	Outcome	Death	Alive	Best C-Statistics	P-value
**SNAP-II	Survival	18 (8.50±)	7.62 (8.8±)	C 0.88 (Snoek 2016) C 0.8 (Our study 2023) C 0.81 (Skarsgard 2005) C 0.79 (Gentili 2015) C 0.79 (Baird 2008) C 0.77 (Coleman 2013)	0.007
***CDHSG-Probability Survival (%)	Survival	65.5 (48-83)	83.5 (76.5-88)	C 0.87 (Gentili 2015) C 0.85 (Baird 2008) C 0.83 (Skarsgard 2005) C 0.77 (Sekhon 2019) C 0.79 (Our study 2023)	0.003
*CDHSG Defect Size	Survival			C 0.9 (Werner 2016) C 0.69 (Our study 2023)	0.307
A		1 (25%)	7 (43.8%)		
B		0	5 (31.2%)		
C		3 (75%)	4 (25%)		
D		0	0		
**Brindle Score	Mortality	2.36 (1.29±)	0.93 (1±)	C 0.80 (Our study 2023) C 0.81 (Brindle 2014) C 0.8 (Clohse 2018) C 0.74 (Bent 2018) C 0.74 (Sekhon 2019)	0.003

## Discussion

Our single-center study estimated the prevalence of CDH to be six per 10,000 (36 inborn) live births between 2000 and 2021 out of 66,844 total births in KAMC. A previous study by Rayes et al. showed a prevalence of one per 2320 live births in King Fahad Hospital (KFH) in Al Baha, which can be explained by the fact that our center is a tertiary center, unlike KFH. Furthermore, the duration of our study covered 22 years, compared to 11 years in Rayes et al. [12].

To our knowledge, our center ranks the highest in terms of CDH prevalence in Saudi Arabia. The mortality rate of CDH in KAMC was estimated to be 51.1%. On the other hand, in Saudi Arabia, several studies reported a mortality rate ranging from 28.6% to 48%, which means that our mortality rate is higher compared to other centers in our region. Moreover, the demographics of CDH patients were closely similar to those of other studies in Saudi Arabia [12-15]. Most cases occur on the left side, with the absence of hernia sac formation and abdominal organs [15-17]. [MSAB1] In the literature on newborns in the United States, the highest prevalence of CDH is in the western region. In the South, African Americans had the lowest prevalence of CDH. Moreover, in the South, the Caucasians had differences between women and men [16]. The mortality rate, according to a study from the United States, was 1183 of the 2356 (51%) newborns with CDH who did not survive. 1052 newborns have not been repaired, and 363 (30.7%) newborns have not had mechanical ventilation [17]. 

Lung-to-head ratio (LHR), an antenatal mortality predictor tool, was found to have a significant difference in terms of p-value. When compared to the literature, this study is the first to report statistical significance in LHR [18,19]. The lung-to-head ratio (LHR) had a high C-statistics (C 0.83), which is consistent with other studies that analyzed LHR as a predictor for the prognosis of CDH antenatally; in addition, our study had the highest C-statistics among other international literature [18,19]. To our knowledge, LHR was mentioned in some studies in Saudi Arabia; however, C-statistics were not calculated.

In our study, stomach and liver herniation were found to be statistically insignificant in predicting survival antenatally, which contradicts what was reported by Basta et al. and Gentili et al. [20,21]. In addition, one study in Saudi Arabia mentioned stomach herniation as a predictor, which does not correlate with our findings [11]. Moreover, contrary to our study, liver herniation was reported in a few studies to be significant, according to a previous meta-analysis [22]. Possible reasons could be due to the low number of cases that were diagnosed antenatally and the low number of cases that presented with stomach or liver herniation.

There were three postnatal predictors that were significant in determining mortality, which corresponds to the literature. This study demonstrates the application of SNAP-II in predicting neonatal survival (C 0.80, p=0.007), which is relatively close to what Snoek et al. reported (C 0.88) [23]. To our knowledge, no other studies in Saudi Arabia have reported the use of SNAP II as a predictor. In addition, the analysis confirms CDHSG-PS as a significant tool in predicting the survival of neonates (C = 0.79, p = 0.003), which goes with what was reported by Sekhon (2019) [24]. No other studies in Saudi Arabia have reported the use of the CDHSG-PS equation as a predictor. CDHSG defect size was proven to be an insignificant predictor (C 0.69, p = 0.307) compared to what Werner et al. reported (C 0.9) [25]. However, the difference in significance could be explained by high mortality, hence the missed chance to report the defect size. To our knowledge, no other studies in Saudi Arabia have reported the use of CDHSG defect size.

In our study, the Brindle Score was approved statistically to be significant in predicting postnatal death (C 0.8, p = 0.003); it was also close to what was reported by Brindle et al. (C 0.81) [26]. No other studies in Saudi Arabia reported the use of the Brindle Score as a predictor. Definitions of mortality predictor tools are mentioned in Appendix 1 [26-30].

One limitation of our study is the biases related to the nature of the study, which depend on the available data and the records reported retrospectively. Missing data could affect our results; however, our findings are consistent with the literature; hence, we think the bias in this matter is minimal. It’s recommended to do a prospective cohort study to draw more accurate conclusions. Moreover, the cases that were referred from outside had minimal data availability in terms of antenatal variables or no data at all, and most were diagnosed incidentally; thus, they presented the mild or no pulmonary hypoplasia group that lived to get transferred. Such a group represents the good prognosis spectrum, while excluding this group from the analysis could lead to more robust results. This could also ameliorate the low sample size in our center.

This study is a retrospective cohort, single-centered study and is the first to calculate predictors of CDH in Saudi Arabia. This study included only 45 neonates, of which 14 had missing data in the predictor tool analysis only, which can create bias in its evaluation and C-statistics; however, the demographic and characteristics analysis were present. Nevertheless, comparing our sample size to other samples in literature made in Saudi Arabia, ours is within average. We recommend further large-scale multi-center, preferably prospective research, be needed to investigate the accuracy of some predictor tools. Regardless, our results indicate the need for the application of the predictor tools in KAMC to help in managing CDH cases and to allocate resources.

## Conclusions

The prevalence of CDH was considered higher compared to international figures. Our mortality rate is comparable to international figures. The most significant predictors of mortality were lung-to-head ratio (prenatally), SNAP-II, CDHSG-probability of survival, and Brindle Score (postnatally). Further multicentered studies are recommended with a larger sample size.

## Appendices

**Table 4:** Definitions of mortality predictor tools %LH: Lung to head ratio, CDHSG: Congenital diaphragmatic hernia study group, SNAP: Scores for neonatal acute physiology, CDH: Congenital diaphragmatic hernia, ECHO: Echocardiogram, MBP: Mean blood pressure, P/F ratio: PaO2/FiO2.

Predictor of mortality Tools	Definition
%LH	It is a sonographic measure proposed to identify fetuses with congenital diaphragmatic hernia (CDH) that have a poor prognosis [26].
CDHSG	The CDHSG score is calculated as follows: 1 (low birth weight) + 1(low Apgar) + 2 (missing Apgar) + 2 (severe pulmonary hypertension) +2 (major cardiac anomaly) + 1(chromosomal anomaly) = Total CDH score [27].
Brindle Score	A total score of 8 that determine the risk of mortality by the following variables (birth weight, 5 min Apgar, pulmonary hypertension, ECHO, Cardiac/Autosomal anomaly) [27].
SNAP-ll	A score that determines the chance of survival by picking the worst of the following variables (MBP, lowest temperature, P/F % ratio, Lowest serum pH, presence of seizure, and urine output during the first 12/24h of life [28].
CDHSG Defect Size	A methos that has 4 subtypes (“A” defects are entirely surrounded by muscle, “B” defects have a small (<50%) and “C” defects large (>50%) portion of the chest wall devoid of diaphragmatic tissue; “D” patients have complete or near absence of diaphragm) to determine the survival rate of neonates.
